# Microbial DNA extraction of high-host content and low biomass samples: Optimized protocol for nasopharynx metagenomic studies

**DOI:** 10.3389/fmicb.2022.1038120

**Published:** 2022-12-21

**Authors:** Polona Rajar, Achal Dhariwal, Gabriela Salvadori, Roger Junges, Heidi Aarø Åmdal, Dag Berild, Drude Fugelseth, Ola Didrik Saugstad, Ulrik Lausten-Thomsen, Gorm Greisen, Kirsti Haaland, Fernanda Cristina Petersen

**Affiliations:** ^1^Department of Neonatal Intensive Care, Division of Paediatric and Adolescent Medicine, Oslo University Hospital Ullevål, Oslo, Norway; ^2^Institute of Oral Biology, Faculty of Dentistry, University of Oslo, Oslo, Norway; ^3^Department of Infectious Diseases, Oslo University Hospital, Oslo, Norway; ^4^Institute of Clinical Medicine, Faculty of Medicine, Oslo University, Oslo, Norway; ^5^Department of Paediatric Research, University of Oslo, Oslo, Norway; ^6^Department of Neonatology, Copenhagen University Hospital Rigshospitalet, Copenhagen, Denmark

**Keywords:** microbiome, whole metagenomic sequencing, host DNA depletion, low biomass, respiratory microbiome, resistome, premature infant, antimicrobial resistance

## Abstract

**Introduction:**

Low microbial biomass and high human DNA content in nasopharyngeal aspirate samples hinder comprehensive characterization of microbiota and resistome. We obtained samples from premature infants, a group with increased risk of developing respiratory disorders and infections, and consequently frequent exposure to antibiotics. Our aim was to devise an optimal protocol for handling nasopharyngeal aspirate samples from premature infants, focusing on host DNA depletion and microbiome and resistome characterization.

**Methods:**

Three depletion and three DNA extraction protocols were compared, using RT-PCR and whole metagenome sequencing to determine the efficiency of human DNA removal, taxonomic profiling and assignment of antibiotic resistance genes. Protocols were tested using mock communities, as well as pooled and individual patient samples.

**Results:**

The only extraction protocol to retrieve the expected DNA yield from mock community samples was based on a lytic method to improve Gram positive recovery (MasterPure™). Host DNA content in non-depleted aliquots from pooled patient samples was 99%. Only samples depleted with MolYsis™ showed satisfactory, but varied reduction in host DNA content, in both pooled and individual patient samples, allowing for microbiome and resistome characterisation (host DNA content from 15% to 98%). Other depletion protocols either retrieved too low total DNA yields, preventing further analysis, or failed to reduce host DNA content. By using Mol_MasterPure protocol on aliquots from pooled patient samples, we increased the number of bacterial reads by 7.6 to 1,725.8-fold compared to non-depleted reference samples. PCR results were indicative of achieved microbial enrichment. Individual patient samples processed with Mol_MasterPure protocol varied greatly in total DNA yield, host DNA content (from 40% to 98%), species and antibiotic resistance gene richness.

**Discussion:**

Despite high human DNA and low microbial biomass content in nasopharynx aspirates of preterm infants, we were able to reduce host DNA content to levels compatible with downstream shotgun metagenomic analysis, including bacterial species identification and coverage of antibiotic resistance genes. Whole metagenomic sequencing of microbes colonizing the nasopharynx may contribute to explaining the possible role of airway microbiota in respiratory conditions and reveal carriage of antibiotic resistance genes.

## Introduction

Sequencing technologies have given us insight into the detailed structure of microbial communities inhabiting various niches of the human body. Evidence that microbiome composition and interactions with host cells influence human physiology and pathology are being increasingly reported in the literature. So far, most studies have focused on the gut microbiome, partly due to its abundance and accessibility. Microbial communities in sites with low microbial biomass such as the nasopharynx, are more challenging and less investigated (Biesbroek et al., 2014). The nasopharyngeal microbiome has the potential to carry implications for disease of upper and lower respiratory tract (Mizgerd, 2014; Teo et al., 2018). Common colonizers of the nasopharynx include species with high pathogenic potential (e.g., *Streptococcus pneumoniae*, *Klebsiella pneumoniae, Staphylococcus aureus*), as well as colonizers that seldom cause diseases, but can serve as a reservoir of antibiotic resistance genes (ARG; Morley et al., 2019; Manenzhe et al., 2020).

Microbiome develops most dynamically in the first 2–3 years of life (Milani et al., 2017). Factors influencing early colonization can carry serious health implications early and later in life (Rautava et al., 2012). Premature infants have immature immune system and are often early exposed to antibiotics, disrupting the developing microbiome, increasing the presence of ARG and thus also contributing to increased antimicrobial resistance in general, one of the main threats to global health (World Health Organization, 2014; Gasparrini et al., 2019). Application of next generation sequencing technology broadens knowledge of the effect of the different variables on the microbiome and resistome, and the discovery of ARG.

The methodological and financial obstacles are substantial in low microbial biomass and high host DNA samples (Shi et al., 2022). Efficient removal of host DNA is necessary before sequencing, to allow for a cost and time efficacy and precise analysis of the samples (Nelson et al., 2019). Furthermore, due to its low microbial biomass, such samples are more subjected to biases or false positives due to contamination during sampling and processing (Salter et al., 2014; Eisenhofer et al., 2019; Douglas et al., 2020). Recently published minimal standards requirements for microbiome studies (Greathouse et al., 2019) should be followed while striving further towards the establishment of generally accepted and applied standard operating procedures for different sample types for human microbiome studies. To date, there is a lack of established standard operating procedures for low biomass samples (Theodosiou et al., 2020; Hasrat et al., 2021).

The aim of this study was to compare the efficiency of different protocols combining host DNA depletion and microbial DNA extraction from nasopharyngeal aspirates of premature infants, for the purpose of microbiome and resistome profiling using whole metagenomic sequencing (WMS).

## Materials and methods

### Ethical statement

The study was performed in accordance with the Declaration of Helsinki and approved by the Hospital’s Data Protection Officer and the Regional Committee for Medical and Health Research Ethics - South East, Norway (2018/1381 REKD), and by the Danish National Committee for Health Research Ethics (H-180512193). Written informed consent was obtained from infant’s parents. The participants received no compensation.

### Samples and experimental design

#### Patient samples

**Figure 1:**
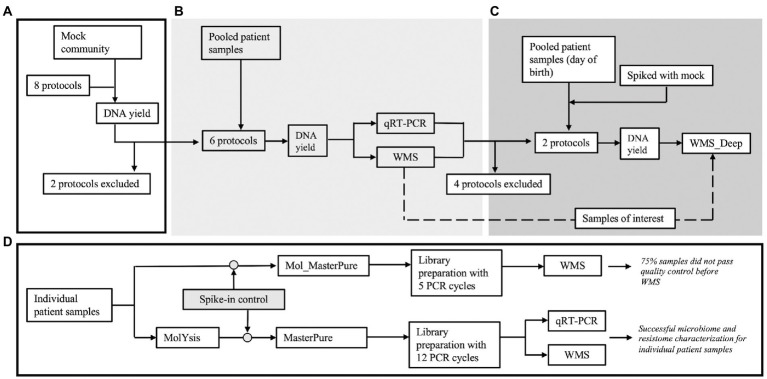
Overview of the experiments. **(A)** Eight protocols were initially tested with Mock community samples (Zymo, D6300) to evaluate loss of DNA during processing. Two protocols were excluded prior to processing clinical samples. **(B)** Three pools (A, B, and C) were created by blindly pooling six 2 ml samples from premature infants for each pool. Six aliquots (2 ml each) from each pool were processed according to the remaining six protocols. **(C)** After reviewing results, four protocols were excluded and an additional pool was created using six samples from premature infants, obtained right after birth (Pool D). Six aliquots (2 ml) from this pool were processed according to the remaining two protocols. Two of the aliquots were spiked with mock community. **(D)** The most promising protocol (Mol_MasterPure) was further tested on individual patient samples.

Nasopharyngeal aspirate samples (n = 42) were obtained from premature infants born between 28^+0^ and 31^+6^ weeks gestational age during their stay at the Neonatal Intensive Care units at Ullevaal, Oslo University Hospital, Oslo, Norway and Rigshopitalet, Copenhagen, Denmark. Samples were obtained by inserting a sterile suction catheter along the nasal wall into the nasopharynx, applying vacuum suction for 5 s and removing the suction catheter without active suction. Standard protection equipment to avoid contamination was used. There was no pre-moisturizing of the suction catheter. A sterile 2 ml 20% glycerol solution was suctioned directly afterwards through the suction catheter to rinse any mucus remains and for cryopreservation of the sample. The samples were rapidly moved to −80°C, where they were stored for up to 10 months. In the laboratory, 18 random samples were divided into Pools A, B, C, each comprising samples from six infants and processed according to different protocols (Table 1). Six samples obtained within 24 h of birth were pooled into Pool D. This experimental design was chosen so that each pool would have sufficient material to be tested with different protocols. Two aliquots from pool D were spiked with mock community (Zymo, D6300) prior to host DNA depletion and DNA extraction to create more diverse samples. Eighteen patient samples were processed individually. They were spiked with Spike-in Control II for Low Microbial Load samples (Zymo, D6321 & D6321-10); 12 samples prior and three post host DNA depletion. Estimated DNA yield of the spike-in was 0.4 ng. The Spike-in Control standard is composed of three species not found in the human microbiome. (*Truepera radiovictrix*, *Imtechella halotolerans* and *Allobacillus halotolerans*). From these, we used *I. haloterans* counts as a reference for quantification of total microbial load. The other two species in the spike-in were excluded from the analysis as they were either non-susceptible to chemical lysis (*T. radiovictrix*) or not in the MetaPhlan database used in the downstream taxonomic analysis (*A. haloterans*). Experimental design is illustrated in Figure 1.

**Table 1:** Host DNA depletion and DNA extraction protocols.

Protocol name	Host DNA depletion kit	DNA extraction kit	Deviation from manufacturer’s protocol
MasterPure	None	MasterPure™ Gram Positive DNA Purification Kit (Epicentre, Madison, WI, United States)	Followed the manufacturer*’*s protocol (Available at: https://www.lucigen.com/docs/manuals/MA209E-MasterPure-Gram-Positive-DNA.pdf)
MagMAX	None	MagMAX™ Microbiome Ultra Nucleic Acid Isolation Kit (Applied Biosystems, Foster City, CA)	We followed the protocol for High throughput isolation of Nucleic Acid (RNA and DNA) from soil, biofluids, and other samples using Bead tubes and the KingFisher™ Duo Prime (Avaliable at: https://www.thermofisher.com/document-connect/document-connect.html?url=https://assets.thermofisher.com/TFS-Assets/LSG/manuals/MAN0018070_MagMAXMicrobiomeNuclAcidIsolatKit_SoilSalivaUrine_Automated_UG.pdf)
QIAamp	None	QiAmp DNA Microbiome Kit (Qiagen, Hilden, Germany)	For DNA extraction, we followed the protocol (Available at: https://www.qiagen.com/us/resources/resourcedetail?id=c403392b-0706-45ac-aa2e-4a75acd21006&lang=en), starting with step 6. (bacterial cells lysis).
PMA_MasterPure	lyPMA	MasterPure™ Gram Positive DNA Purification Kit	We followed the published method protocol for host DNA depletion with lyPMA (Marotz et al., 2018). DNA extraction was performed as described in MasterPure protocol.
PMA_MagMax	lyPMA	MagMAX™ Microbiome Ultra Nucleic Acid Isolation Kit	We followed the published method protocol for host DNA depletion with lyPMA (Marotz et al., 2018). DNA extraction was performed as described in MagMax protocol.
Mol_MasterPure	MolYsis™ Basic5 (Molzym, Bremen, Germany)	MasterPure™ Gram Positive DNA Purification Kit	We followed the manufacturer*’*s protocol (Available at: http://www.goffinmoleculartechnologies.com/wp-content/uploads/2012/01/MolYsis_Basic5_V3.0.pdf) for 1 ml samples and accordingly doubled the volume of reagents used in points 1. and 2. DNA extraction as described in MasterPure protocol.
Mol_MagMax	MolYsis™ Basic5	MagMAX™ Microbiome Ultra Nucleic Acid Isolation Kit	We followed the manufacturer*’*s protocol (Available at: http://www.goffinmoleculartechnologies.com/wp-content/uploads/2012/01/MolYsis_Basic5_V3.0.pdf) for 1 ml samples, and accordingly doubled the volume of reagents used in steps 1. and 2. DNA extraction as described in MagMax protocol.
QIA_QIAamp	QIAamp	QIAamp DNA Microbiome Kit	We followed the protocol (Available at: https://www.qiagen.com/us/resources/resourcedetail?id=c403392b-0706-45ac-aa2e-4a75acd21006&lang=en) for 1 ml samples and accordingly doubled to volume of the reagent used in step 1.

#### Positive and negative control

Mock community samples used for positive control were prepared to match the low concentration of DNA found in nasopharyngeal aspirate samples from premature infants measured in a pilot study (data not shown). Two microliter of mock community (Zymo, D6300) with expected DNA yield of approximately 55 ng were diluted in 2 ml of sterile 20% glycerol solution, to resemble the preparation of patient samples. The samples were placed on ice and processed immediately. All experiments with mock community samples were run in triplicates.

The cryoprotectant (2 ml sterile 20% glycerol solution, prepared in sterile conditions) used for clinical samples was vacuum suctioned into a sterile mucus trap at the NICU, under the same conditions as when obtaining samples from the infants, and later processed with each extraction method, serving as a control for contamination during its production and the sampling procedure. Reagent controls (for each used extraction kit) were extracted with each extraction method and served as controls for kit contamination. The negative controls had too low concentration to be used for library prep and were excluded from further processing.

### Host DNA depletion and DNA extraction methods

Samples were processed with combinations of different depletion and extraction methods (Table 1). The starting volume for all samples was approximately 2 ml, and the final DNA elution volume 30–50 μl. Samples underwent no additional freeze–thaw cycle prior to completed DNA extraction. The amount of extracted DNA was measured using Qubit™ dsDNA HS kit, on a Qubit 2.0 Fluorometer (Life Technologies, Darmstadt, Germany) and NanoDrop™ spectrophotometer (Thermo Fisher Scientific, Waltham, MA, United States).

### Real time PCR

Human DNA was amplified using the primer pair FP1065 5’ GCCCGTTCAGTCTCTTCGATT and FP1066 5’ CAAGGCAAAGCGAAATTGGT for the RPL30 gene, and bacterial DNA using the 16S rRNA universal primers FP1067 5’ CCATGAAGTCGGAATCGCTAG and FP 1068 5’ GCTTGACGGGCGGTGT (Yigit et al., 2016). All reactions were performed in duplicates. The final PCR reaction volume was 25 μl, comprising 12.5 μl Maxima SYBR Green/ROX qPCR Master Mix (2×) containing Maxima Hot Start Taq DNA Polymerase, dNTPs and SYBR Green I in an optimized PCR buffer with ROX passive reference dye, 1 μl DNA template (up to 70 ng), 0.4 μM forward and reverse primers, 1× SYBR green (Life Technologies), and the remainder nuclease-free water. The amplification was carried out over 40 cycles (30 s at 98°C, 60 s at 55°C, 60 s at 72°C) with an initial 10 min hot start at 95°C. Bacterial enrichment was calculated as relative values after normalizing all the data against human DNA and comparing it to non-enriched samples.

Additionally, Femto™ Quantification kits for host and bacterial DNA (Zymo, E2005 and E2006) were used according to the manufacturer’s instructions for four individual patient samples. Used sample volume was 1 μl.

### Library preparation

Nextera DNA Flex Library Prep Kit (Illumina Inc., San Diego, CA, United States) was used for library preparation, following manufacturer’s protocol. The only deviation was initially using five PCR amplification cycles for all library preparations (against producers’ recommendations of 12 PCR cycles for low DNA input), to reduce bias and enable comparison between samples. However, individual patient samples retrieved very low DNA yields and only three of the first 12 samples passed quality control and were sequenced. To optimize this step, we increased the PCR cycle number to 12 for six additional samples and used DNA input comparable to the DNA yield of the first 12 samples (6 ng). Library concentration and purity were measured with Qubit™ dsDNA HS kit on a Qubit 2.0 Fluorometer (Life Technologies, Darmstadt, Germany), NanoDrop™ spectrophotometer (Thermo Fisher Scientific, Waltham, MA, United States) and Bioanalyzer 2,100 (Agilent, Santa Clara, CA, United States).

### Sequencing

WMS was conducted at the Norwegian Sequencing Centre (Oslo, Norway). WMS was run on an NovaSeq SP platform (Illumina Inc., San Diego, CA, United States) using a paired-end sequencing approach with a targeted read length of 125 bp in high-output mode.

### Data analysis

Quality of raw reads was assessed using FASTQC (Andrews, 2010). Adapter sequences and low-quality reads were removed with Trimmomatic (Bolger et al., 2014). Further on, filtered quality reads were aligned to human reference genome using Bowtie2 (Langmead and Salzberg, 2012) in order to remove human DNA contamination. The remaining high quality clean reads were used for microbiome and resistome profiling. Microbiota profiling was done with Metaphlan3 (Beghini et al., 2021). For the resistome analysis, the quality-filtered, clean reads were provided as input to Bowtie2 (Langmead and Salzberg, 2012) alignment using default parameters to the ResFinder database (Florensa et al., 2022). Reads were assigned to ARGs using an 80% gene coverage/fraction threshold. Counts of reads aligned to the ARGs were then used for downstream comparative analyses.

### Rarefaction analysis

We performed rarefaction analysis to estimate the required sequencing depth needed to characterize microbiome and resistome at various taxonomic levels. Seqtk tool (Li, 2012) was used to sample clean reads into subsamples at various depths (10, 25, 30 50, 75% etc.), followed by taxonomic profiling using Metaphlan 3 (Beghini et al., 2021) to report the number of species present within each subsample. RarefactionAnalyzer tool of the AMRPlusPlus pipeline (Doster et al., 2020) was used with 5% subsampling increments of the read data with 10 iterations at each level for resistome rarefaction analysis. The numbers of unique species, genes, mechanisms, and classes were plotted as a function of sampling depth using the ggplot2 package in R (Wickham, 2016).

## Results

### DNA recovery from low biomass microbial mock samples

**Figure 2:**
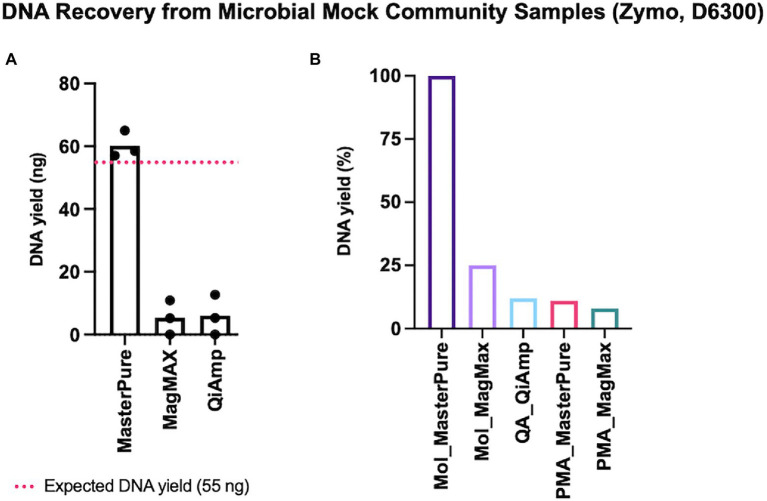
DNA recovery from mock samples. **(A)** Three commercial DNA extraction kits without depletion steps were tested. Estimated input was 55 ng (dashed red line). Values for individual samples are presented as dots. Bars correspond to mean values from three independent experiments. **(B)** Retrieved DNA yield using protocols composed of host DNA depletion and DNA extraction steps. Values were normalized to the mean yield obtained with Mol_MasterPure protocol. Bars represent retrieved mean DNA yield relative to the mean yield obtained with Mol_MasterPure protocol.

Some microbial DNA may be lost during both host DNA depletion and DNA extraction procedures. We explored this using low concentrations of a defined microbial mock community with an expected DNA yield of 55 ng (2 μl, Zymo D6300). Of the three different commercial kits for microbial DNA extraction, the approximate expected yield was recovered only with MasterPure protocol (Figure 2A). We continued with testing protocols that deplete host DNA pre-extraction using 2 μl of Zymo mock community. Among the five protocols with DNA depletion, recovery was highest with Mol_MasterPure protocol, which retrieved on average (SD) 23.5 (9.7) ng DNA. Four other protocols with depletion retrieved on average less than 25% of DNA yield, relative to the yield obtained with Mol_MasterPure protocol (Figure 2B).

### DNA recovery from patient samples and evaluation of microbial enrichment through real-time qPCR

**Figure 3:**
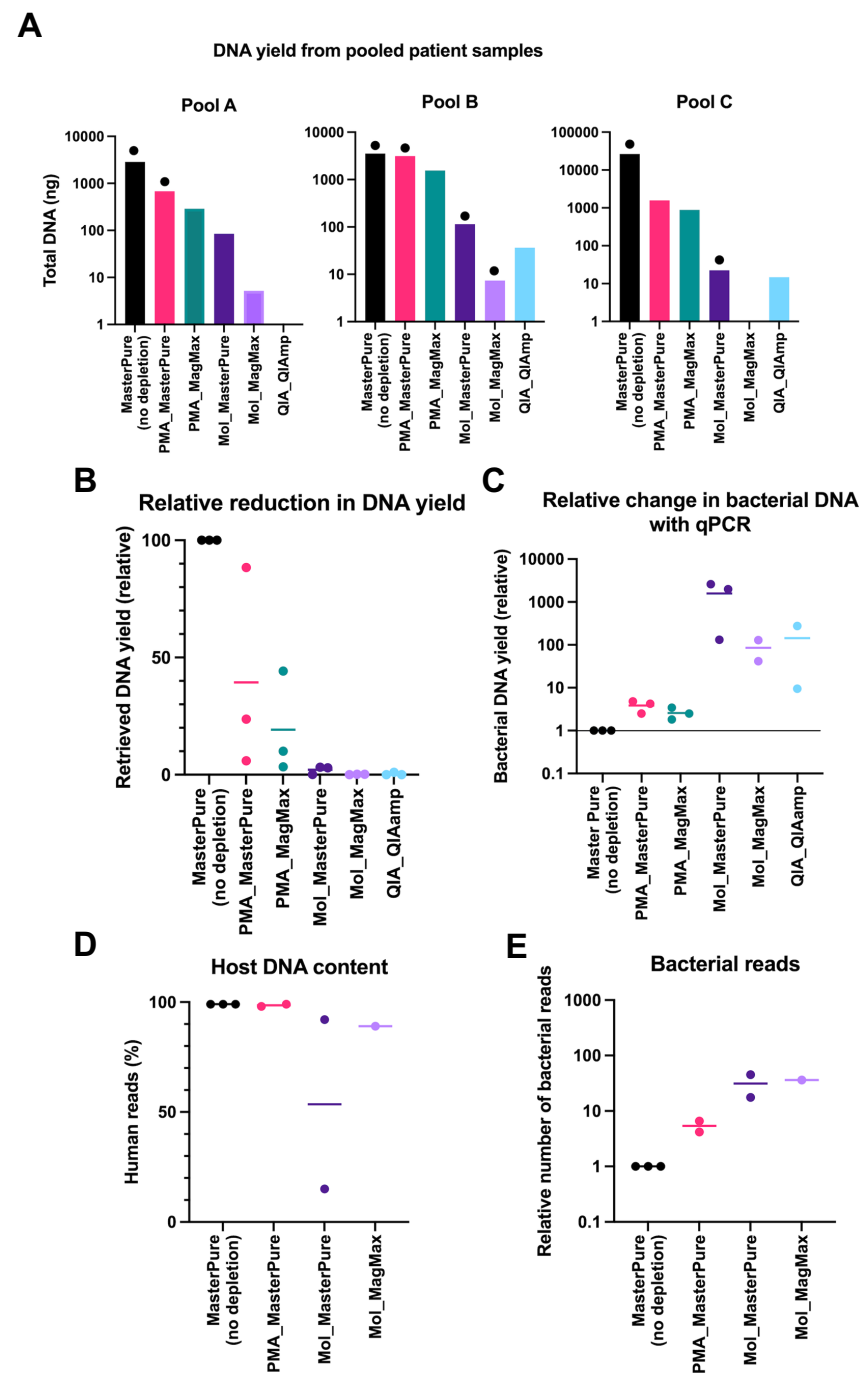
Pooled patient samples. **(A)** DNA yield extracted from pools A, B, and C according to different protocols. Each bar represents DNA yield of one sample. Samples that proceeded to WMS are marked with black dots (library concentration > 10 nM). **(B)** Comparison of relative reduction in total DNA yield with protocols for DNA depletion. MasterPure (no depletion) was used as a reference. **(C)** Relative change in the proportion of microbial DNA/host DNA, evaluated with real-time qPCR. **(D)** Host DNA content evaluated with WMS in samples from patients’ pools A, B, and C. MasterPure protocol served as a reference. **(E)** Increase in number of bacterial reads in depleted samples, relative to non-depleted reference samples. **(B–E)** Values for individual samples are presented as dots. Bars correspond to mean values from samples from different patient pools, processed with the same protocol.

To test the performance of the different combinations of depletion and extraction methods in human low biomass samples, nasopharynx aspirates from premature infants were blindly grouped into three pools (A, B, C), each comprising samples from 6 different infants. MasterPure, the protocol that retrieved the highest DNA amount using mock communities (Figure 2A) served as a non-depletion reference protocol to which the other protocols were compared to. All protocols that included a host DNA depletion step showed a reduction in total DNA recovery compared to the no depletion reference (Figure 3A). Sixteen samples from pools A, B and C were evaluated with real time qPCR to determine whether the proportion of microbial DNA/host DNA increased following DNA depletion. Two samples (QA_QIAamp from pool A and Mol_MagMax from pool C) were excluded due to low DNA yield. Protocols using lyPMA for depletion recovered on average more than 19% of DNA yield (Figure 3B) and showed the poorest performance in microbial DNA enrichment (Figures 3C–E) under tested conditions. Lower recovery of total DNA and higher enrichment were seen with protocols using MolYsis or QIAamp for depletion.

### Evaluation raw reads with whole metagenome sequencing at increasing sequencing depth

Sixteen samples from patient pools A, B and C with DNA concentration above 0.01 ng/μl were further processed for library preparation. Eight of the 16 samples produced libraries with concentration > 10 nM, minimum threshold recommended by the sequencing provider, and underwent WMS performed at an average depth of 15 M reads per sample. Detailed information regarding library preparation and raw reads are listed in Table 2. Information regarding excluded samples is listed in the Online Supplement (Supplementary Table 1). In reference samples (no depletion, MasterPure protocol) from all three pools the percentage of reads belonging to host DNA was 99%. All six samples processed with QIA_QIAamp and PMA_MagMax were excluded prior to WMS (Supplementary Table 1). Five samples processed with other host DNA depletion protocols passed the criteria to proceed to WMS (Table 2). Their host DNA content is shown in Figure 3D. From Pool D, all six aliquots were further processed for library preparation. One sample was excluded prior to WMS (Supplementary Table 1). Sequencing depth was an average of 54 M reads per sample (Protocol Name_Deep). Detailed information regarding library prep and raw reads are listed in Table 2.

**Table 2:** Information regarding library preparation, host DNA content and number of bacterial, and ARG associated reads.

Pool	Protocol name	Library DNA input (ng)	Library conc. (ng/𝜇l)	Molarity pool sent for seq. (nM)	Total reads	Preprocessed reads	Human reads		Remaining reads		Bacterial reads	Bacterial reads (% of Remaining reads)	ARG richness (No of ARG)	ARG associated reads	ARG associated reads (% of Remaining reads)
Pool A	MasterPure (no depletion)	499.4	14.6	10	15.83 M	12.57 M	12.44 M	99%	0.13 M	1.0%	0.02 M	14.8%	3	178	0.10%
	PMA_MasterPure	408	4.7	10	15.63 M	9.01 M	8.84 M	98%	0.17 M	1.9%	0.11 M	62.0%	8	612	0.40%
	Mol_MasterPure_Deep*	50.7	0.1	0.4 (low)	11.12 M	7.46 M	1.43 M	19%	6.04 M	80.9%	5.49 M	90.9%	20	90,326	1.50%
Pool B	MasterPure (no depletion)	501.3	16.6	10	15.86 M	12.38 M	12.27 M	99%	0.11 M	0.9%	0.00 M	1.4%	0	0	0.00%
	PMA_MasterPure	499.2	4	10	13.31 M	9.87 M	9.75 M	99%	0.12 M	1.2%	0.01 M	9.5%	0	0	0.00%
	Mol_MasterPure	68.4	5.7	10	17.74 M	12.88 M	11.79 M	92%	1.09 M	8.5%	0.71 M	65.2%	8	780	0.10%
	Mol_MasterPure_Deep	68.4	5.7	6	66.89 M	51.11 M	46.68 M	91%	4.43 M	8.7%	2.87 M	64.7%	12	4,699	0.10%
	Mol_MagMax	4.4	3.9	10	15.30 M	13.22 M	11.80 M	89%	1.42 M	10.7%	0.74 M	52.2%	11	1,318	0.10%
Pool C	MasterPure (no depletion)	530	15.6	10	14.43 M	12.77 M	12.66 M	99%	0.11 M	0.9%	0.00 M	4.2%	0	0	0.00%
	MasterPure_Deep (no depletion)	530	15.6	6	66.71 M	61.59 M	61.07 M	99%	0.52 M	0.8%	0.02 M	4.7%	0	0	0.00%
	Mol_MasterPure	13.4	3.2	10	15.62 M	13.17 M	2.03 M	15%	11.14 M	84.6%	8.19 M	73.5%	32	49,578	0.40%
	Mol_MasterPure_Deep	13.4	3.2	6	67.95 M	58.12 M	8.73 M	15%	49.38 M	85.0%	36.91 M	74.7%	32	225,237	0.50%
Pool D	MasterPure.1_Deep (no depletion)	502.2	22.8	6	54.27 M	49.44 M	49.04 M	99%	0.41 M	0.8%	0.02 M	4.1%	0	0	0.00%
	MasterPure.2_Deep (no depletion)	177.6	17.8	6	54.29 M	49.43 M	49.02 M	99%	0.41 M	0.8%	0.01 M	2.1%	0	0	0.00%
	Mol_MasterPure.1_Deep	18.4	0.8	6	44.53 M	29.51 M	29.01 M	98%	0.49 M	1.7%	0.13 M	25.5%	13	1,350	0.30%
	Mol_MasterPure.1_Deep, spiked	19.9	0.5	6	53.49 M	28.06 M	9.97 M	36%	18.10 M	64.5%	17.02 M	94.0%	8	67,411	0.40%
	Mol_MasterPure.2_Deep, spiked	103.8	1.4	6	63.94 M	54.21 M	23.39 M	43%	30.82 M	56.9%	28.02 M	90.9%	17	55,989	0.20%

Rows in grey correspond to parallel samples that have been sequenced deeper. The initial threshold for sending libraries for sequencing was reduced from 10 to 6 nM for the samples with the lowest biomass, pool D, to enable inclusion of all five samples at equal quantities in the sequencing pool. *Sample Mol_MasterPure from pool A had too low concentration to be included in the equimolar pool but was added to the second sequencing round at a lower concentration.

### Impact of different methods and sequencing depth on microbiome profile characterization

Three samples processed with the protocol Mol_MasterPure, which showed promising results with regards to host DNA removal, and one reference sample (Pool C) were additionally sequenced with increased depth (approximately 54 M reads per sample; Protocol Name_Deep, Figure 1C) to explore the influence of sequencing depth on the recovered bacterial reads and ARGs (Table 2).

**Figure 4:**
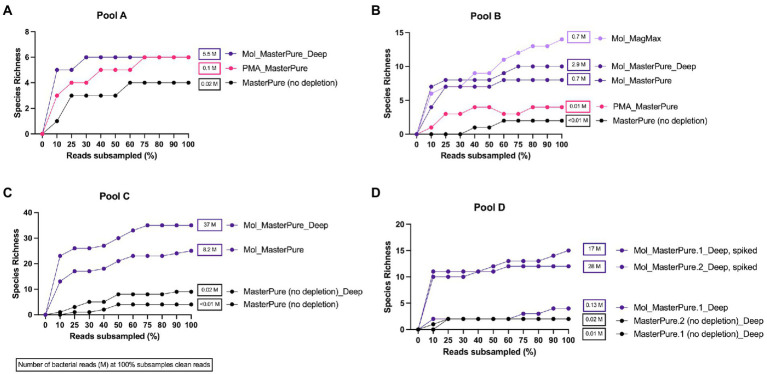
Rarefaction analysis. Rarefaction analysis. Rarefaction curves at species level for samples at various sequencing depth (% clean reads) for pools A **(A)**, B **(B)**, C **(C)**, and D **(D)**. Protocols including MolYsis for host DNA depletion retrieved highest species richness across all pools. Number of bacterial reads obtained at 100% clean reads are listed next to samples rarefaction curve.

**Figure 5:**
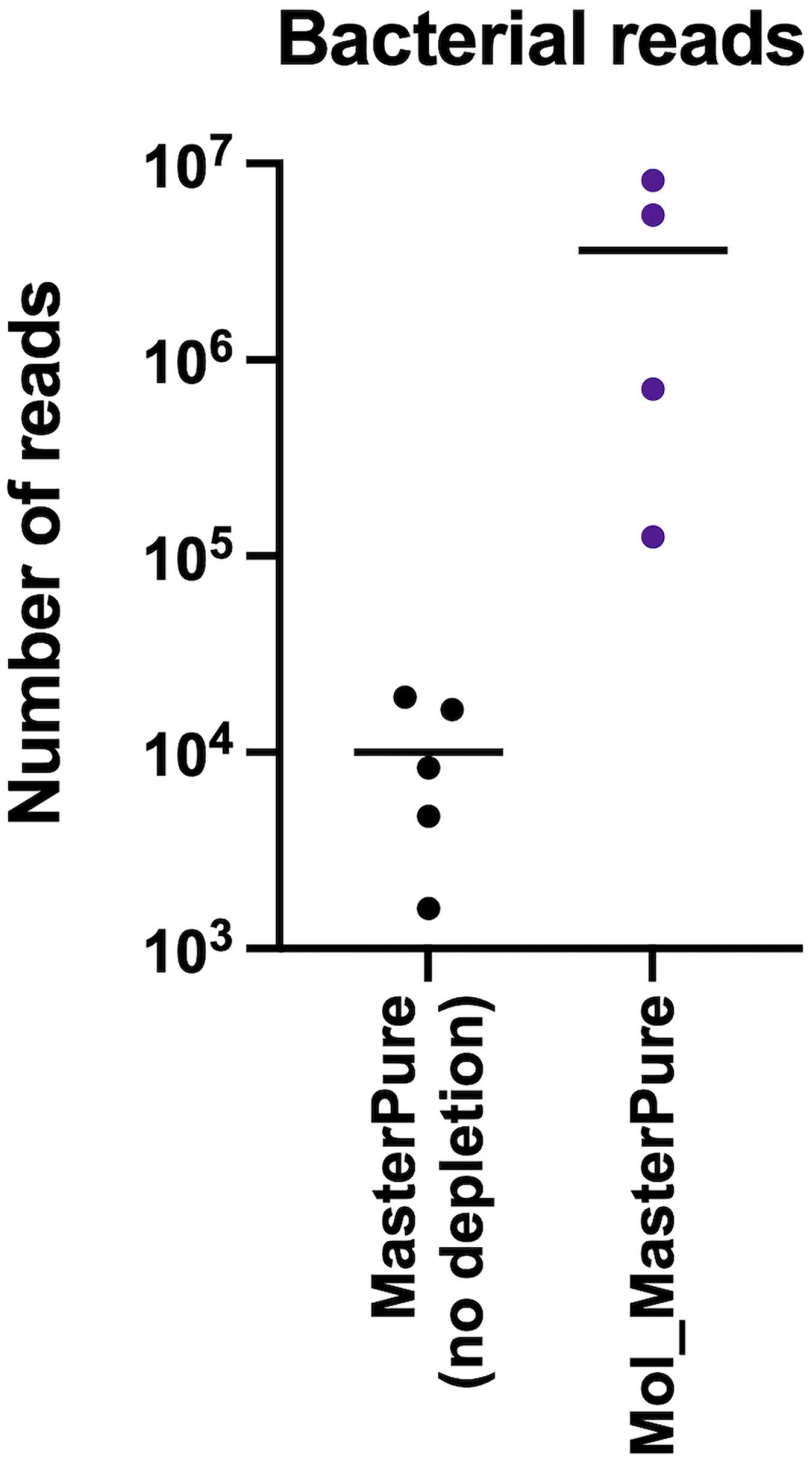
Bacterial reads after processing with MolYsis. Number of reads assigned to bacteria in non-depleted reference samples and in depleted samples using the Mol_MasterPure protocol from the four pools (A, B, C, D). The values for each individual samples are presented as dots. The horizontal lines correspond to mean values.

Rarefaction analysis was performed to investigate whether enough bacterial reads were obtained to represent the species richness in each of the samples (Figure 4). Mol_MasterPure protocol preformed best across all patient pools, compared with other protocols. Sufficient sequencing depth to characterize the microbiome on species level was obtained also from two samples processed with PMA_MasterPure (Pool A) and Mol_MagMax protocol (Pool B; Figure 4). From pool D, only the spiked aliquots (Mol_MasterPure, spiked) showed a greater reduction in the host DNA content allowing to capture full diversity of species according to the rarefaction analysis (Figure 4). Merged data from both WMS rounds, comparing reference (MasterPure) and Mol_MasterPure protocols, showed that the Mol_MasterPure protocol resulted in (mean, range) 495.6 (7.6 to 1,725.8) -fold increase in the number of bacterial reads (Figure 5).

Relative abundance (Supplementary Figure 1) remained similar when the same samples were analysed after shallow and deep WMS, on genus and species level. No visible differences in the taxonomic composition were observed between aliquots processed with different protocols. From pool D, only the two aliquots spiked with Zymo mock (D6300) passed the rarefaction analysis (Supplementary Figure 1).

### Effect of host DNA content and sequencing depth on resistome characterization

From the 17 samples sent to WMS, ARG could be assigned from datasets of nine samples. Eight of these samples were depleted with MolYsis, and two (Mol_MasterPure sample from pool B and C) were sequenced at two different sequencing depths.

**Figure 6:**
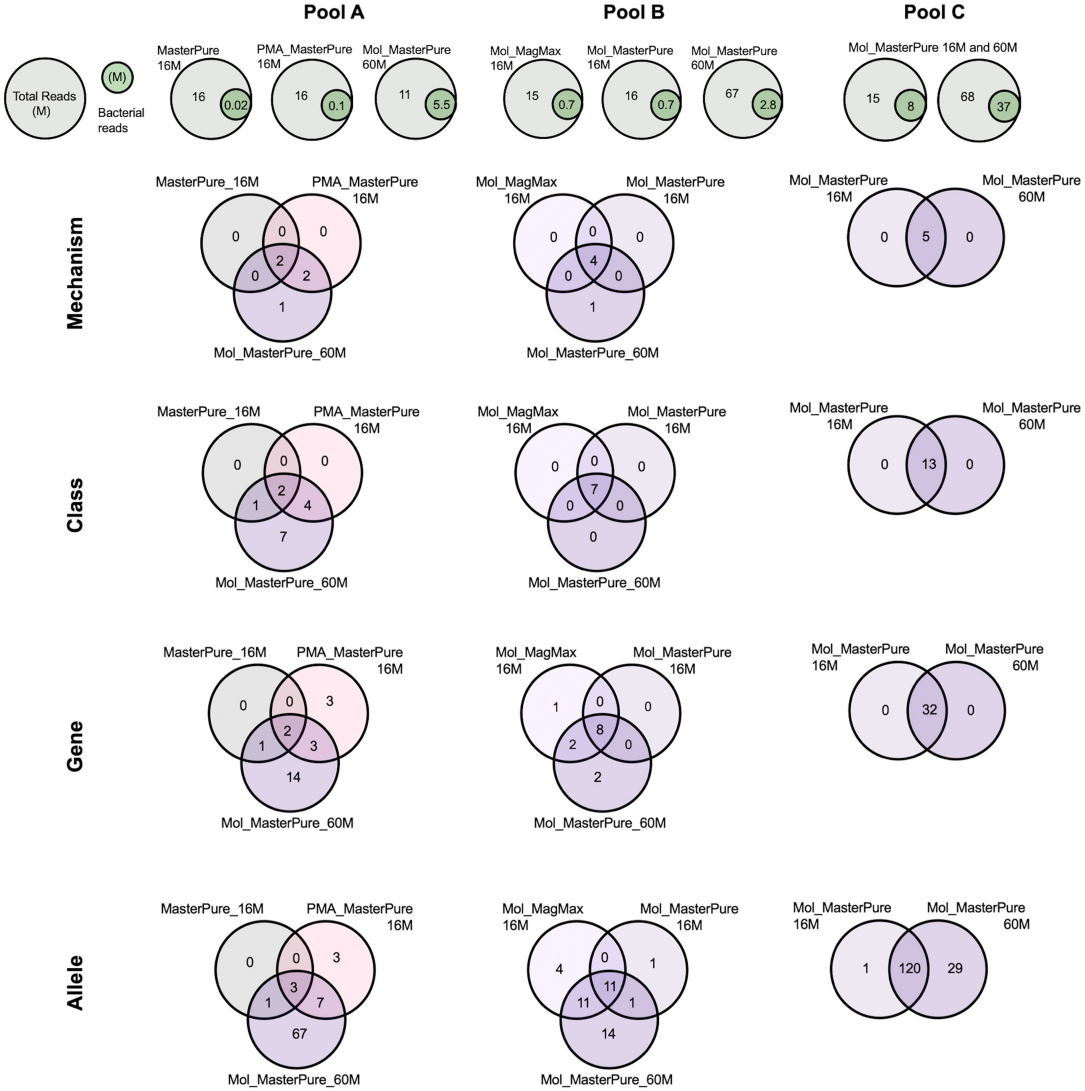
Venn diagrams representing intersection of different resistome annotation levels (Mechanism, Class, Gene, Allele) for patient samples from pools A, B, and C, processed according to different protocols and sequenced at different sequencing depths.

All three samples originating from pool A, processed with different protocols, had a similar number of total reads (11–16 M reads), but differed in host DNA content and consequently number of bacterial reads (Table 2). The Mol_MasterPure aliquot had the lowest host DNA content (19%) and the highest number of unique antimicrobial resistance (AMR) determinants on all annotation levels (Figure 6). The two samples from pool B had equal number of total reads (16 M) and similar host DNA content (Mol_MP 92%, Mol_MM 89%). Increasing the sequencing depth of Mol_MasterPure to 67 M reads resulted in detection of unique AMR determinants. Sample Mol_MasterPure from pool C had the lowest host DNA content (15%) and was sequenced at two sequencing depths. Obtaining 68 M vs. 16 M total reads resulted in detection of 29 unique AMR determinants on allele level, with no changes on mechanism, class and ARG level. Increase of AMR determinants at various resistome classification levels resulting from both reduction in host DNA content and increase in sequencing depth is shown in Figure 6. The resistome composition at different annotation levels is shown in Supplementary Figure 2.

**Figure 7:**
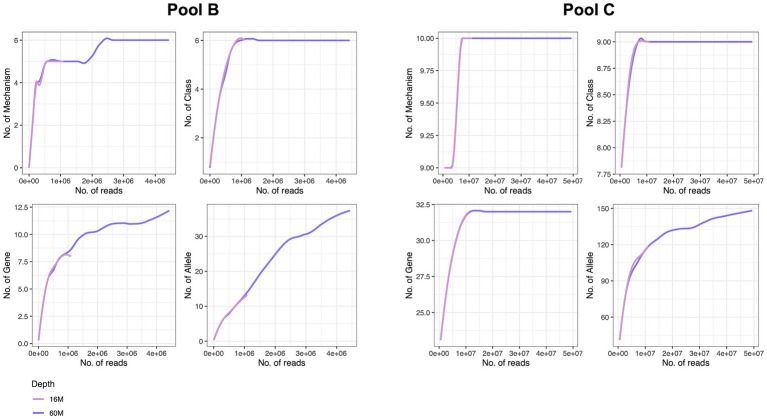
Rarefaction analysis of two samples with different host DNA content at two sequencing depths at mechanism, class, gene and allele levels, performed on remaining reads (after removal of host reads). Both samples were processed with Mol_MasterPure protocol. Host DNA content was 92% for the sample originating from pool B, and 15% for the sample originating from pool C. Number of total reads for sample from pool B processed with Mol_MasterPure protocol was 18 M (pink line) and 68 M (purple line). Number of reads assigned to bacteria was 0.7 M and 2.9 M, respectively. Number of total reads for sample from pool C processed with Mol_MasterPure was 15 M (pink line) and 68 M (purple line). Number of reads assigned to bacteria was 8.2 M and 27 M, respectively.

To determine the necessary sequencing depth for resistome characterisation we further performed a rarefaction analysis at different annotation levels for two samples (Mol_MasterPure) originating from different patient pools (B and C) sequenced at two sequencing depths. Samples from pool B still presented high host DNA content (92%) after depletion with MolYsis. The increase in sequencing depth improved resistome characterization, reaching saturation at mechanism and class levels, but the rarefaction curves at gene and allele levels still did not appear to have reached the plateau (Figure 7). Samples from pool C had a lower host DNA content. The rarefaction analysis for mechanism, class and gene reached the saturation plateau already at sequencing depth of 16 M. Increasing the sequencing depth to 68 M improved the resolution on allele level as well, but the rarefaction analysis suggests further increase in sequencing depth might increase the number of characterized alleles (Figure 7).

### Individual patient samples

**Figure 8:**
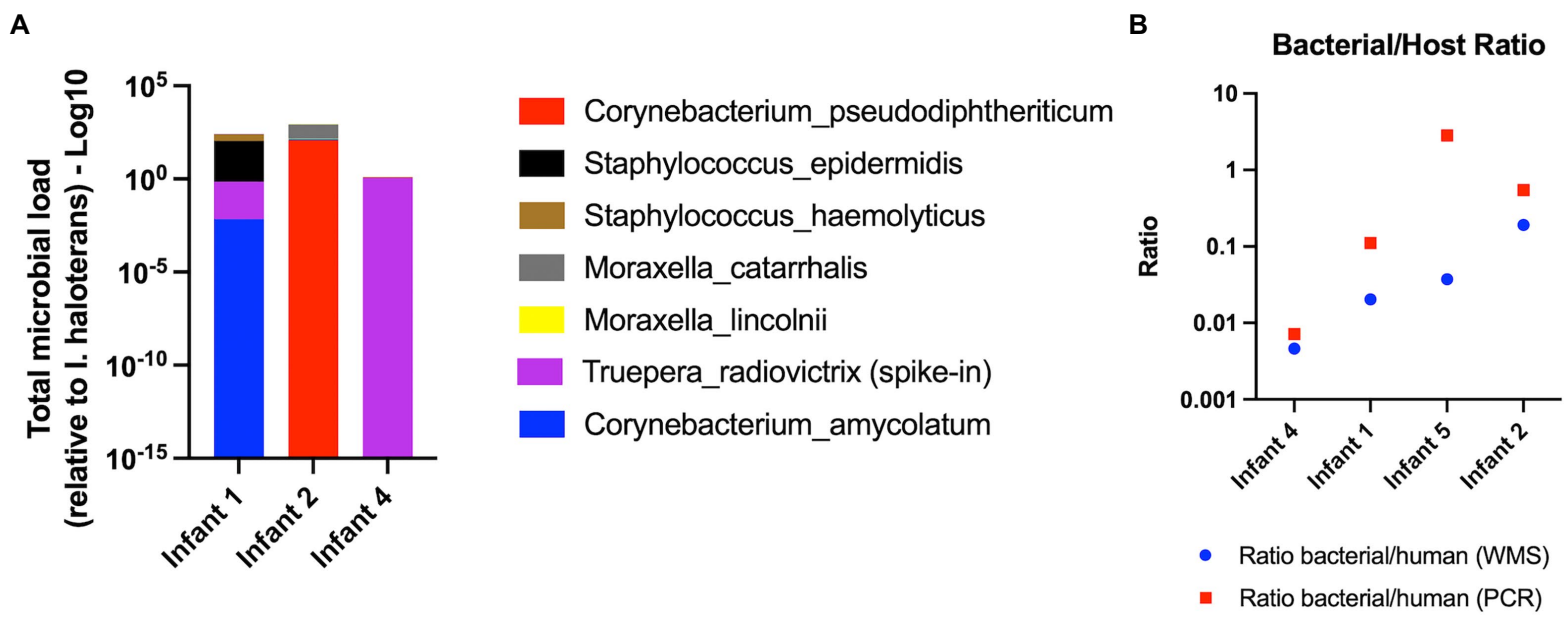
Individual patient samples. **(A)**. Total microbial load relative to the abundance of I. haloterans (log scale). **(B)** Bacterial/Host DNA ratio for individual patient samples evaluated with real time qPCR (red) and WGS (blue).

We further tested the Mol_MasterPure protocol on 18 patient samples obtained at timepoints from birth to 6 months corrected age, to explore how the protocol performs despite the variations expected in individual samples (Figure 1D). Twelve samples prepared identical to the pooled samples, using five PCR cycles during library preparation, had very low yield after library preparation (median, range: 6.48 ng, 5.88–123 ng). Nine of these 12 samples failed quality control prior to WGS. Six samples prepared using 12 PCR cycles (DNA input 6 ng) produced libraries of sufficient concentration and quality and were sequenced at an average depth of 32 M reads (details regarding library preparation, the number of initial, human and bacterial reads are listed in Table 3). Relative abundance of classified bacterial taxa, together with rarefaction analysis can be found in the online supplement (Supplementary Figure 3). Three samples were spiked with a standardized Zymo Spike-in Control II (Zymo, D6321 & D6321-10) after host DNA depletion. Their total microbial load (relative to *I. haloterans*) is shown in Figure 8A. To observe a possible correlation between host DNA content obtained with real time qPCR and WGS, four samples were also quantified using Femto quantification kit for bacterial and host DNA (Figure 8B).

**Table 3:** Detailed information on individual patient samples.

Sample name	DNA quantity (ng)	Library DNA input (ng)	Library conc. (ng/𝜇l)	Molarity pool sent for seq. (nM)	Total reads	Preprocessed reads	Human reads		Remaining reads		Bacterial reads	Bacterial reads (% of Remaining reads)	ARG richness (No of ARG)	ARG associated reads	ARG associated reads (% of Remaining reads)
Infant 1*	195.6	6.5	28.2	25	35.23 M	35.23 M	33.40 M	95%	1.83 M	5.2%	0.68 M	37.0%	11	8,624	0.47%
Infant 2*	714	6	11.2	25	22.98 M	22.98 M	15.89 M	69%	7.09 M	30.9%	3.04 M	42.9%	5	10,980	0.15%
Infant 3	6.8	6.6	15.7	25	46.24 M	46.24 M	18.69 M	40%	27.55 M	59.6%	12.33 M	44.8%	5	33,812	0.12%
Infant 4*	44.1	6.3	6.6	25	26.70 M	26.70 M	26.04 M	98%	0.66 M	2.5%	0.12 M	18.1%	0	0	0%
Infant 5	199.2	6	17.6	25	25.38 M	25.38 M	23.66 M	93%	1.72 M	6.8%	0.88 M	51.2%	12	131,665	7.65%
Infant 6	6.8	6.6	10.5	25	37.63 M	37.63 M	36.62 M	97%	1.01 M	2.7%	0.02 M	1.5%	2	90	0.01%

Six individual patient samples were processed with Mol_MasterPure protocol. Samples from Infant 1, Infant 4, Infant 5, were from infants between 0 and 7 days old, and samples from Infant 2 and Infant 3 between 56 days and 6 months old. *Samples were spiked with 20 𝜇l (0.4 ng) of Zymo Spike-in Control II for Low Microbial Load samples (Zymo, D6321, and D6321-10) after host DNA depletion.

## Discussion

We performed a method optimization study for low microbial biomass samples with high human DNA content for the purpose of microbiome and resistome characterization with WMS using nasopharyngeal aspirates from premature infants. We found that nasopharynx aspirates of preterm infants have a high host DNA content (99%). Of the protocols tested in our study, Mol_MasterPure (composed of host DNA depletion with MolYsis™ Basic5 and DNA extraction with MasterPure™ Gram Positive DNA Purification Kit) was the most promising protocol for microbiome and resistome characterization with WMS, tested with pooled and individual patient samples.

Microbial DNA may be partially lost during both host DNA depletion and DNA extraction procedures. This is particularly critical in samples with low microbial biomass. We explored this using a defined microbial mock community. Of the three DNA extraction kits only MasterPure retrieved the expected DNA yield, thus becoming a reference. Host DNA depletion processes remove all extracellular DNA. Since the D6300 mock is stored in RNA/DNA shield, some of the bacterial cells will be lysed prior to processing. Hence, the retrieved DNA yield or the retrieved microbial composition could not be compared with the non-depleted reference.

**Figure 9:**
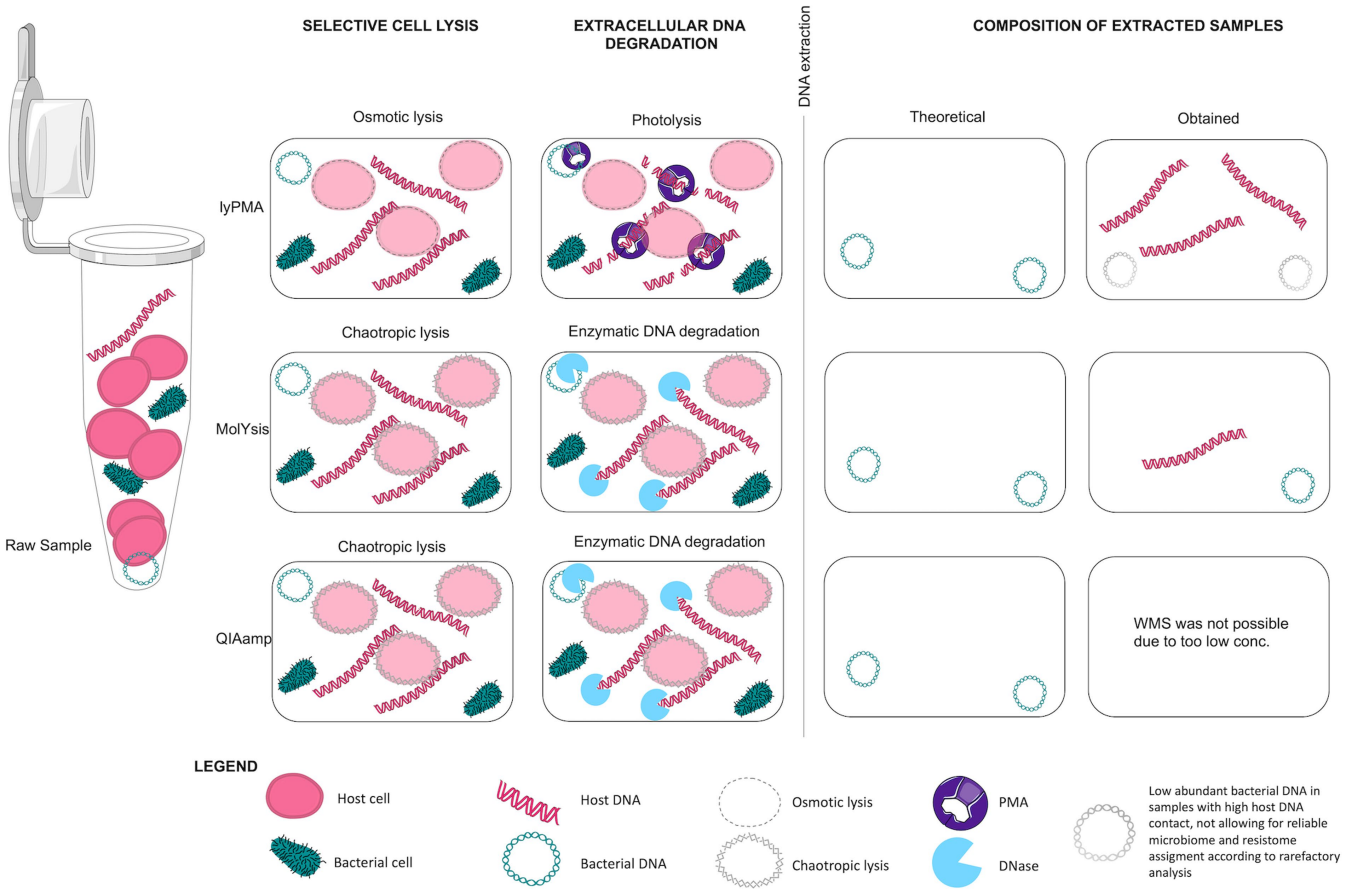
Mechanism of Host DNA depletion protocols. Graphical illustration of different host DNA depletion protocols according to their mechanism of action. LyPMA protocol is composed of osmotic lysis and DNA fragmentation with photolysis. MolYsis and QIAamp use chemical lysis of host cells followed by enzymatic degradation of extracellular DNA. In theory, all methods result in selective lysis of human cells, followed by removal of extracellular DNA, human and bacterial. In our study, only samples depleted with MolYsis™ showed a reduction in host DNA content. Parts of the figure were drawn by using pictures from Servier Medical Art by Servier (smart. servier.com).

Three pre-extraction host DNA depletion methods with selective lysis of human cells and extracellular DNA degradation (Figure 9) were compared in five protocols with pooled patient samples. These depletion methods were chosen as they have been shown to be superior to other pre- and post-extraction host DNA removal methods (e.g., filtration and selective removal of CpG-methylated host DNA) in studies analysing samples with high host DNA content (Thoendel et al., 2016; Marotz et al., 2018; Heravi et al., 2020; Rubiola et al., 2020).

Previously, the lyPMA protocol was found to be more effective than both MolYsis and QIAamp protocols in host DNA removal from saliva samples (Marotz et al., 2018). LyPMA has the advantage of lower costs and short handling time. However, the combination of osmotic lysis and DNA fragmentation with photolysis did not work as efficiently in our experiments. Host DNA content remained as high as in the reference samples indicating that method performance could depend on the sample type. A study using bovine milk samples (also low biomass, with high host DNA content) reported similar results to ours after lyPMA treatment of the samples, even after optimising lyPMA concentration from 10 to 20 μM (Ganda et al., 2021). QIAamp depletion protocol works through a similar mechanism as MolYsis (chemical lysis of host cells and enzymatic degradation of extracellular DNA) and has previously shown to outperform MolYsis in host DNA removal for some samples (Marotz et al., 2018; Heravi et al., 2020). However, in our study, further analysis of the samples processed with QIAmp protocol failed due to too low DNA yield. A threshold of 1 pg DNA/μl for microbiota detection has previously been proposed (Biesbroek et al., 2012). We therefore decided to exclude samples with <1 pg. Although MolYsis was initially developed to selectively isolate and purify bacterial DNA from whole blood samples in aid of sepsis diagnosis (Gebert et al., 2008; Horz et al., 2008), its efficiency in host DNA removal has broadened its use to samples of different origin for microbiome and resistome studies (Hasan et al., 2016; Rubiola et al., 2020; Yap et al., 2020).

Patient samples were initially pooled rather than processed individually, as we expected a large variation between individual samples, potentially preventing us from comparing different processing methods. Variation in species and ARG richness was seen also between the pools. As the samples were blindly pooled, no metadata was collected to supplement the interpretation of our results. We found that nasopharyngeal aspirate samples from premature infants contained a high content of host DNA and removing host DNA with MolYsis prior to DNA extraction was the only successful method for enriching microbial fraction sufficiently for both microbiome and resistome analysis of WMS data. Due to the variation between patient pools and the small number of samples processed with each protocol sent to WMS, no meaningful statistical tests could be implemented to compare host DNA depletion efficiency of different protocols.

High host DNA content interferes with the sensitivity of WMS for taxonomic profiling, even at greater sequencing depths (Pereira-Marques et al., 2019). In addition, methods that amplify specific sequences, such as qPCR and 16S rRNA sequencing, have been demanding to implement for low biomass samples with high host DNA content. A previous study by Gallacher et al. (2020) in a cohort of premature infants showed that only 6.7% of nasopharyngeal aspirate samples obtained in the first 3 days after birth had a high enough bacterial load for 16S amplicon sequencing. Similar low bacterial load was found in some of our samples, reflecting the very low biomass in samples obtained soon after birth.

The most promising protocol Mol_MasterPure was further applied also to individual patient samples. Despite variations in composition between individual samples, all samples met the yield and quality parameters recommended for WMS. Individual patient samples analysed in our study showed variation in obtained DNA yield (Table 3) and host DNA content (from 40 to 98%; details in Table 3). Samples collected in the first week after birth had a higher host DNA content despite host DNA depletion processing, while samples obtained later in life showed greater enrichment. This is expected due to rapid microbial colonization and increase in microbial density of nasopharyngeal microbiome from birth on (Theodosiou et al., 2020).

To evaluate microbial enrichment of our samples prior to WMS and to assist in the estimation of required sequencing depth, we performed real-time qPCR using primers targeting the 16S gene. The microbial enrichment seen with qPCR was indicative of the extent of host DNA removal seen with WMS (Figures 3C, 5). Relative qPCR could serve as a time and cost-efficient triage prior to WMS. However, this would require having a reference sample (without depletion) for every patient sample, which might not be feasible in practice. Alternatively, targeted qPCR absolute quantification methods for both bacterial and host DNA can be used to predict library composition for WMS and help determine needed sequencing depth (Cho et al., 2021). In our individual patient samples, 2 out of 6 samples did not yield sufficient material for both WMS library preparation and two qPCR reactions. The ratio of bacterial / host WMS reads and bacterial / host DNA quantity (qPCR) for the remaining four samples showed some correlation (Figure 8B), but more samples would be required to suggest a possible prediction model.

Equimolar library pooling is necessary to obtain comparable number of total reads for all submitted samples and is preferred over equal-volume pooling for use in patient derived samples with multiple bacterial species (Muller et al., 2019). We initially set the threshold to 10 nM for samples sent to WMS and successfully adjusted it to 6 nM (Pool D; Table 2) due to lower yield from samples obtained within 24 h after birth.

One of the limitations of WMS is providing only relative information on microbial composition. Spiking samples with known absolute abundance serves as a positive control, and additionally enables quantification of microbiome composition (Figure 8A) and contributes towards more unbiased interpretation of dynamics and interactions in the microbiome (Wang et al., 2021). Therefore, spike with known absolute abundance can be helpful in microbiome studies where determining total microbial load is relevant to the aims of the study (Stämmler et al., 2016).

Methods without extracellular DNA removal might overestimate the bacterial composition in analysed patient samples (DNA from viable and non-viable bacteria), restricting interpretation of a possible taxonomic bias between depleted and reference samples. It is however a concern that besides removing extracellular DNA from unviable cells, DNA from bacteria with a thin or missing cell wall could also be lost during host DNA depletion steps (Horz et al., 2010). This could introduce a taxonomic bias especially as a loss of Gram-negative species (Horz et al., 2010; Heravi et al., 2020; Rubiola et al., 2020), as we observed in our samples from Pool D that were spiked with mock community (Zymo D6300) (Supplementary Figure 1). Even though the respiratory tract is mainly colonised by Gram-positive bacteria, some Gram-negative bacteria are also relevant, including for instance *Moraxella* (Gram-negative) (Toivonen et al., 2021). We were not able to obtain enough bacterial reads for microbiome and resistome classification from any of the reference (non-depleted) samples to be able to compare them with their depleted parallels. Our study was, however, not designed for bias analysis. Further, our study’s limitations were using a mock (Zymo, D6300) stored in RNA/DNA shield that could cause cell lysis prior to processing, and not using the producer’s recommended amount of mock community since we aimed to have a better representation of the low biomass of our samples of interest. To address this problem, a mock with viable bacterial cells from cultures of species commonly found in the respiratory microbiome should be created, for a detailed investigation of the possible bias introduced with host DNA depletion protocols. This was not feasible in our study.

In this study we describe how different protocols for host DNA depletion and DNA extraction performed on mock community standards, pooled and individual patient samples from nasopharyngeal aspirates of premature infants. Microbiome and resistome composition from low biomass samples with high host DNA content was best characterized applying a protocol combining depletion with MolYsis™ and extraction with MasterPure™. Analysis of samples obtained immediately after birth remains challenging, and our protocol should be further tested and optimized in settings of a larger study. Our findings may contribute to broadening and improving use of WMS in respiratory and other low biomass microbiota studies.

## Data availability statement

The datasets presented in this study can be found at: https://www.ncbi.nlm.nih.gov/bioproject/PRJNA876384.

## Ethics statement

The studies involving human participants were reviewed and approved by Hospital’s Data Protection Officer and the Regional Committee for Medical and Health Research Ethics–South East, Norway (2018/1381 REKD), and by the Danish National Committee for Health Research Ethics (H-180512193). Written informed consent to participate in this study was provided by the participants’ legal guardian/next of kin.

## Author contributions

All authors contributed to the design of the study. PR, UL-T, and KH collected the samples. PR, GS, and HÅ carried out the laboratory experiments. PR and AD carried out the data analysis. PR wrote the manuscript. KH and FP supervised the overall study. All authors discussed the results, critically revised the manuscript, and agreed to the published version of the manuscript.

## Funding

This work was supported by the Norwegian Research Council (NFR) project number 273833, by Olav Thon Foundation, by the Faculty of Dentistry at the University of Oslo and by Oslo University Hospital.

## Conflict of interest

The authors declare that the research was conducted in the absence of any commercial or financial relationships that could be construed as a potential conflict of interest.

## Publisher’s note

All claims expressed in this article are solely those of the authors and do not necessarily represent those of their affiliated organizations, or those of the publisher, the editors and the reviewers. Any product that may be evaluated in this article, or claim that may be made by its manufacturer, is not guaranteed or endorsed by the publisher.
