# Supplementary material for: Microbial DNA extraction of high-host content and low biomass samples: Optimized protocol for nasopharynx metagenomic studies
Source: Front Microbiol. 2022 Dec 21;13:1038120. doi: 10.3389/fmicb.2022.1038120 (PMC9811202; doi:10.3389/fmicb.2022.1038120)
Supplement: Supplementary file 1 [file Data_Sheet_1.docx]

Supplementary Material

# Supplementary Figures and Tables

**Supplementary Table 1.** **Samples excluded prior to WMS**. Nine samples from Pools A, B, C and D were excluded after library prep due to too low library concentrations. The initial threshold for sending libraries for sequencing was reduced from 10 to 6 nM for the samples with the lowest biomass, pool D, to enable inclusion of all five samples at equal quantities in the sequencing pool. *Calculated library concentration from sample Mol_MasterPure from pool A was <10 nM, but we still included it in the deeper WMS round. ** Sample QIA_QIAamp from pool A and sample Mol_MagMax from pool C were excluded after DNA extraction due to too low concentration (unmeasurable with Qubit^TM^ dsDNA HS kit). *** Sample Mol_MasterPure.2_Deep from Day 0 Pool proceeded to library prep even though its concentration was too low, but was excluded prior to WMS due to too low library concentration (unmeasurable with Qubit^TM^ dsDNA HS kit).

| **Pool** | **Protocol Name** | **Input DNA Library Prep (ng)** | **Library conc. (ng/𝜇l)** | **Calculated library concentration (nM)** |
| --- | --- | --- | --- | --- |
| **Pool A** | PMA_MagMax | 173.4 | 2.4 | 7.5 |
|  | Mol_MasterPure* | 50.7 | 0.1 | 0.4 |
|  | Mol_MagMax | 3.1 | 1 | 3.2 |
|  | QIA_QIAamp | *NA*** | | |
| **Pool B** | PMA_MagMax | 499.2 | 1.6 | 4.8 |
|  | QIA_QIAamp | 21.9 | 0.5 | 1.9 |
| **Pool C** | PMA_MasterPure | 499.3 | 3 | 8.2 |
|  | PMA_MagMax | 500.2 | 0.7 | 2.1 |
|  | Mol_MagMax | *NA*** | | |
|  | QIA_QIAmp | 8.8 | 1.9 | 5.5 |
| **Pool D** | Mol_MasterPure.2_Deep | NA*** | NA*** | NA*** |

**Supplementary Figure 1.** Microbiome composition in samples processed according to different protocols, analysed with WMS at two sequencing depths. Numbers of total reads, reads assigned to bacteria: **(A)** Pool A: PMA_MP 16M, 0.1M; Mol_MP_Deep 11M, 5.492M. **(B)** Pool B: Mol_MP 18M, 0.708M; Mol_MP_Deep: 67M, 2.881M; Mol_MP_Deep: 67M, 2.881M; Mol_MM: 15M, 0.738M. **(C)** Pool C: Mol_MP 15M, 8.246M; Mol_MP_Deep 68M, 37.037M. **(D)** From pool D, only the two aliquots spiked with Zymo mock (D6300) passed the rarefaction analysis (Mol_MP_Mock.1_D and Mol_MP_Mock.2_D). Their composition was dominated by some of the added mock genera (*Bacillus, Listeria, Lactobacillus, Enterococcus, Staphylococcus).* However, the Gram-negative genera from mock were almost completely absent from the processed samples, with no reads assigned to *Pseudomonas* (expected relative abundance 4.2%), <0.0001% of reads assigned to *Salmonella* and <0.001% to *Escherichia* (expected relative abundance 10.4% and 10.1%).

**Supplementary Figure 2. Resistome from pooled patient samples.** RPKM assigned at different annotation levels. **(A)** Mechanism, **(B)** Class, **(C)** Gene and **(D)** Allele from pooled patient samples processed according to different protocols.  Numbers of total reads, reads assigned to bacteria: Pool A: MasterPure_16M: 16M, 0.02M; PMA_MasterPure_16M: 16M, 0.1M ;Mol_MP_60M: 11M, 5.492M. Pool B: Mol_MagMax_16M: 15M, 0.738M, Mol_MasterPure 16M, 0.708M; Mol_MasterPure_60M: 67M, 2.881M. Pool C: Mol_MasterPure _16M: 15M, 8.246M; Mol_MP_60M: 68M, 37.037M. Pool D: Mol_MasterPure_60M: 45M, 0.1M, Mol_MasterPure_Mock.1_D: 53M, 17M; Pool D: Mol_MasterPure_Mock.2_D: 64M, 28M.

**Supplementary Figure 3. Individual patient samples.** Microbiome composition at **(A)** genus and (**B)** species level for individual patient samples processed with Mol_MasterPure protocol. Samples from Infant 1, 2 and 4 were spiked with microbial community standard. We were able to retrieve two species from the spike-in control in all three samples (Imtechella haloterans and Trupera radiovicitrix), which together represented 90% relative abundance in sample from infant 4, but their relative abundance was too low to be visible in the figure of samples from infant 1 and 2. (**C)** Rarefactory analysis for individual patient samples at bacterial species level, and class, ARG and allele resistome levels.
