# Supplementary figures and images for: Microbial DNA extraction of high-host content and low biomass samples: Optimized protocol for nasopharynx metagenomic studies
Source: Front Microbiol. 2022 Dec 21;13:1038120. doi: 10.3389/fmicb.2022.1038120 (PMC9811202; doi:10.3389/fmicb.2022.1038120)

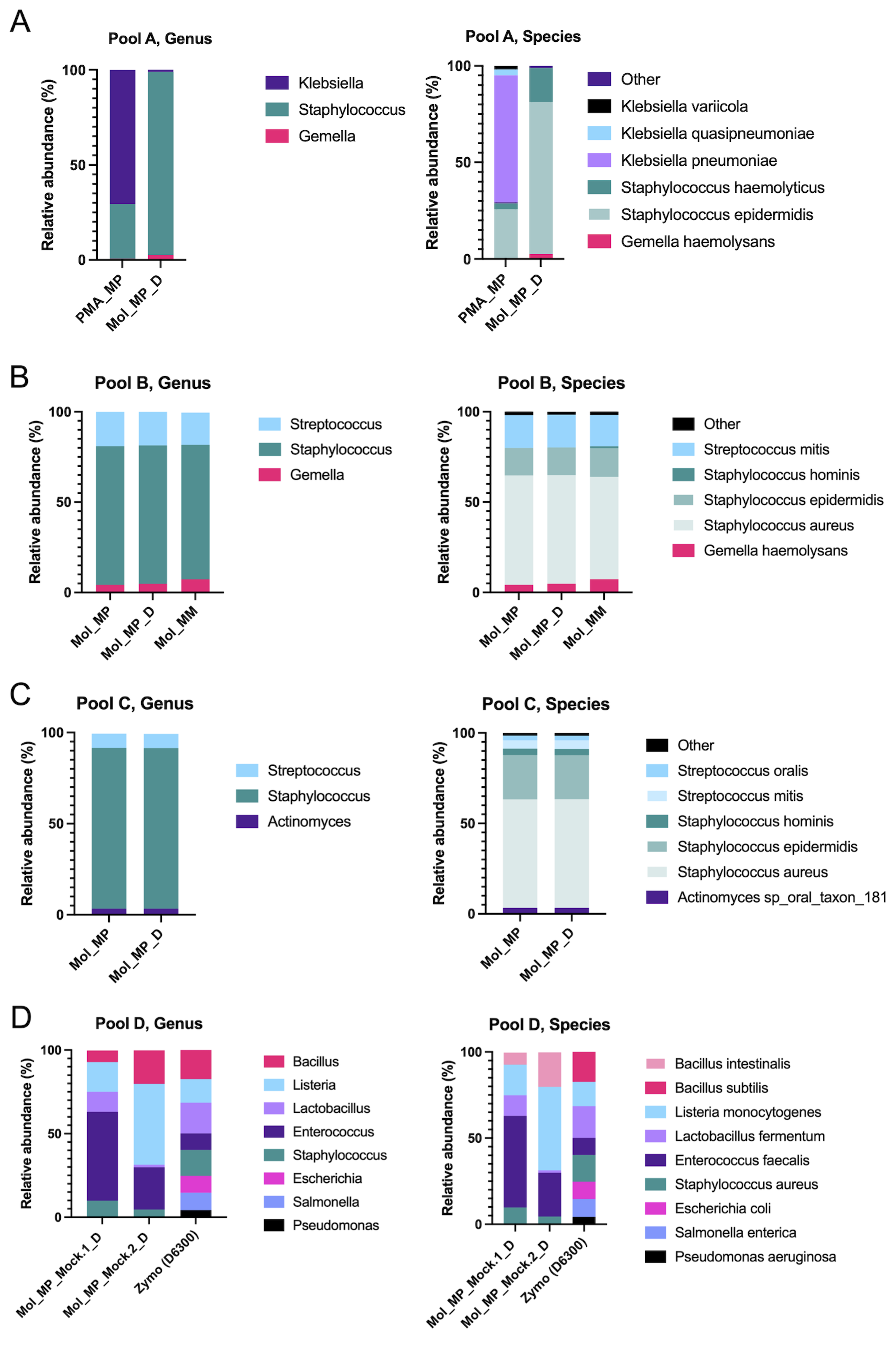

Supplement: Supplementary file 2 [file Image_1.TIFF]

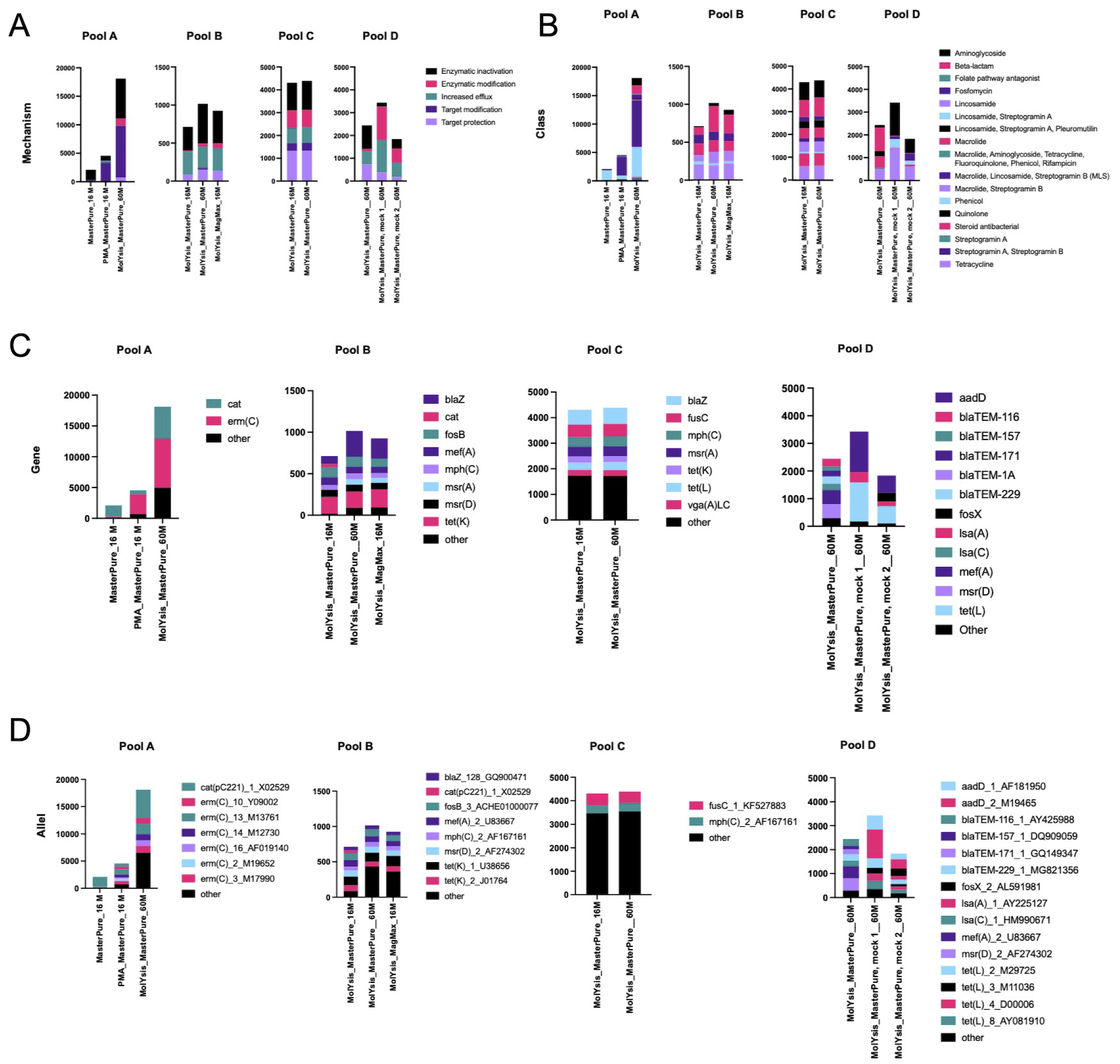

Supplement: Supplementary file 3 [file Image_2.TIFF]

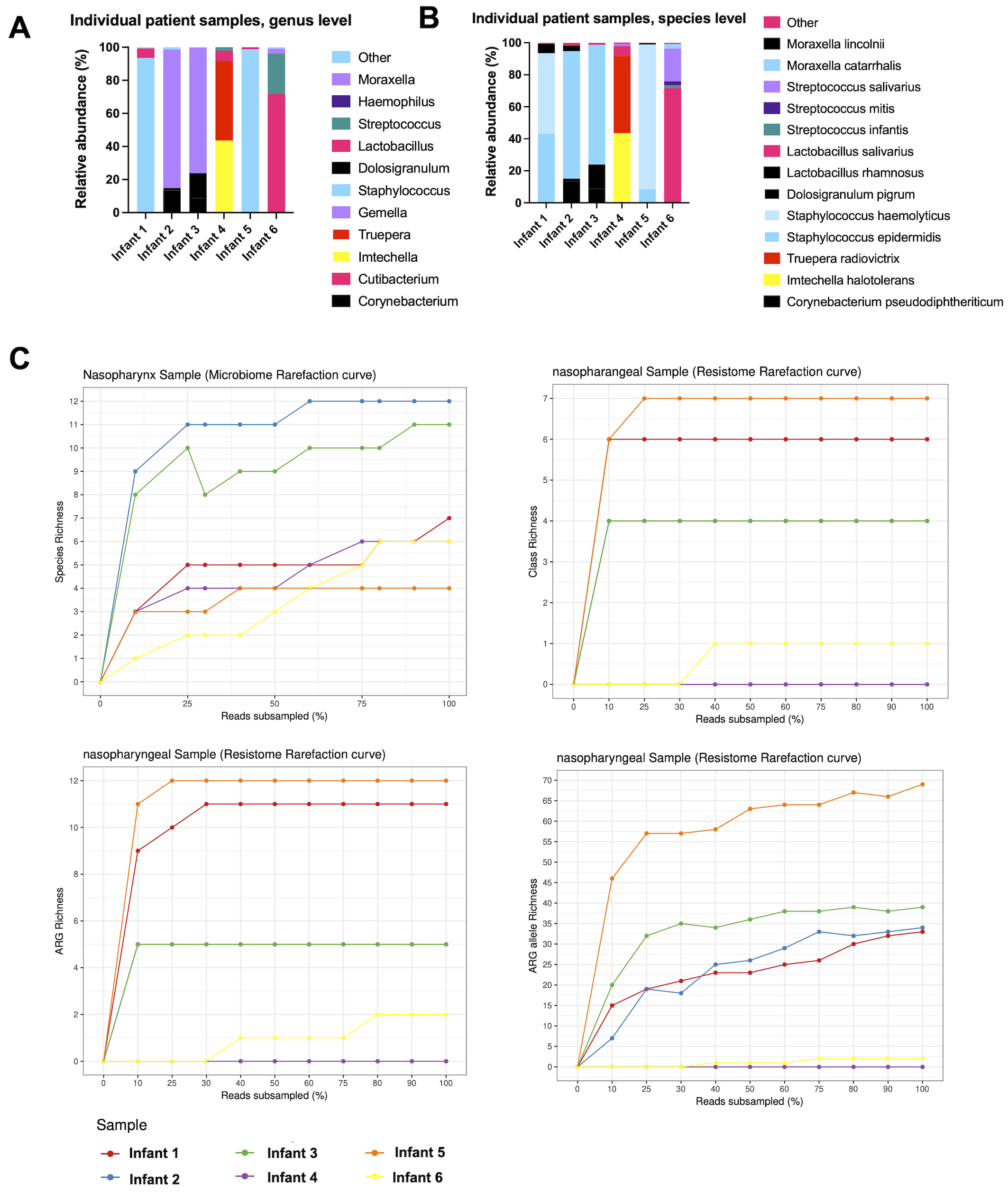

Supplement: Supplementary file 4 [file Image_3.TIFF]
